# The History and Science of the Major Birch Pollen Allergen Bet v 1

**DOI:** 10.3390/biom13071151

**Published:** 2023-07-19

**Authors:** Heimo Breiteneder, Dietrich Kraft

**Affiliations:** Division of Medical Biotechnology, Department of Pathophysiology and Allergy Research, Center of Pathophysiology, Infectiology and Immunology, Medical University of Vienna, 1090 Vienna, Austria; dietrich.kraft@aon.at

**Keywords:** Bet v 1, major birch pollen allergen, molecular allergology, PR-10-like protein family, allergen ligands

## Abstract

The term allergy was coined in 1906 by the Austrian scientist and pediatrician Clemens Freiherr von Pirquet. In 1976, Dietrich Kraft became the head of the Allergy and Immunology Research Group at the Department of General and Experimental Pathology of the University of Vienna. In 1983, Kraft proposed to replace natural extracts used in allergy diagnostic tests and vaccines with recombinant allergen molecules and persuaded Michael Breitenbach to contribute his expertise in molecular cloning as one of the mentors of this project. Thus, the foundation for the Vienna School of Molecular Allergology was laid. With the recruitment of Heimo Breiteneder as a young molecular biology researcher, the work began in earnest, resulting in the publication of the cloning of the first plant allergen Bet v 1 in 1989. Bet v 1 has become the subject of a very large number of basic scientific as well as clinical studies. Bet v 1 is also the founding member of the large Bet v 1-like superfamily of proteins with members—based on the ancient conserved Bet v 1 fold—being present in all three domains of life, i.e., archaea, bacteria and eukaryotes. This suggests that the Bet v 1 fold most likely already existed in the last universal common ancestor. The biological function of this protein was probably related to lipid binding. However, during evolution, a functional diversity within the Bet v 1-like superfamily was established. The superfamily comprises 25 families, one of which is the Bet v 1 family, which in turn is composed of 11 subfamilies. One of these, the PR-10-like subfamily of proteins, contains almost all of the Bet v 1 homologous allergens from pollen and plant foods. Structural and functional comparisons of Bet v 1 and its non-allergenic homologs of the superfamily will pave the way for a deeper understanding of the allergic sensitization process.

## 1. Events That Led to the Discovery of the Major Birch Pollen Allergen Bet v 1

Clemens von Pirquet, a true legend of immunology [1], observed that antibodies were not only part of protective immune responses but could also cause diseases. He coined the word “allergy,” derived from ancient Greek (allos = other and ergon = work), to generally describe a change in the reactive capability of the immune system. He presented his findings in 1906 in the Münchener Medizinische Wochenschrift, thus introducing the term “allergy” to the medical terminology.

In 1976, Dietrich Kraft [2] became the head of the Allergy and Immunology Research Group at the Department of General and Experimental Pathology, then of the Medical Faculty of the University of Vienna, which would become the Medical University of Vienna in 2004. In autumn 1983, Kraft turned his full focus to allergic diseases. Working in an outpatient allergy clinic in Vienna together with his colleague Herwig Ebner, he came to the insight that allergy test solutions and hyposensitization solutions could only be standardized based on pure recombinant molecules. Kraft elaborated on this idea during a lengthy stay in hospital due to a severe bicycle accident. Consequently, on 12 December 1983, he called Michael Breitenbach on the phone, as a meeting in person was impossible due to the unusually heavy snowfall that day. During this one hour call, Kraft managed to persuade Michael Breitenbach to contribute his essential expertise in molecular cloning to this newly hatched project. Thus, the keystone for the founding of the Vienna School of Molecular Allergology was put into place. Kraft also recruited Otto Scheiner and Helmut Rumpold, who brought additional expertise, i.e., immunohistochemistry and medical laboratory techniques, to the project.

From 1983 to 1984, investing in the cloning and production of recombinant allergens was of no interest for funding agencies in Austria. In a similar manner, pharmaceutical companies active in the allergy field in countries including Denmark, Sweden, Germany, and the USA did not support Kraft’s plans, deeming them to be too exotic. During a banquet held by the Austrian Society of Allergology and Immunology (ÖGAI), Kraft ended up sitting next to Jörg Mayrhofer, to whom he laid open his vision for recombinant allergens. Mayrhofer, then owner of the Schutzengel Apotheke (Guardian Angel Pharmacy) in Linz, Austria, showed an interest in Kraft’s plans as a potential business opportunity. During a follow-up visit to Linz, Jörg Mayrhofer and his father, Theodor Mayrhofer, agreed to fund Kraft’s ambitious plans. Consequently, in 1984, the company Biomay was founded that would finance all the formative years of Kraft’s team of molecular allergology.

As around 5% of the Austrian population suffer from pollinosis induced by birch pollen in early spring, and as there is only one major allergen present in birch pollen extract, this allergen was chosen as the target. Following the rules of allergen nomenclature laid down by the WHO/IUIS Allergen Nomenclature Sub-Committee [3] and the then-valid taxonomical name for the white birch, *Betula verrucosa*, this allergen was designated as Bet v 1. Michael Breitenbach selected Heimo Breiteneder as the person who would be responsible for the cloning of the cDNA for Bet v 1. Breiteneder had just finished his thesis on the genome organization of the cyanelles of *Cyanophora paradoxa* [4] under the guidance of Wolfgang Löffelhardt at the Institute of General Biochemistry of the University of Vienna.

On 2 May 1985, the work on the cloning of the Bet v 1 cDNA started in earnest. At first, the isolation of RNA from birch tissues needed to be established. This was done from roots of birch seedlings, and from leaves and inflorescences of mature trees. Finally, pure pollen from birch trees was obtained, RNA was isolated and poly(A)^+^ mRNA was enriched. This mRNA preparation was translated in vitro in a cell-free wheat germ system, and the proteins synthesized were separated by SDS-PAGE and transferred to nitrocellulose. When the blots were incubated with sera from patients allergic to birch pollen, IgE binding to a 17-kD protein, presumably Bet v 1, was observed, indicating the presence of allergen-coding mRNA in the RNA preparations [5]. Based on this finding, the *E. coli* phage λgt11 was selected to produce a cDNA expression library from birch pollen poly(A)^+^ mRNA. Michael Breitenbach was also instrumental in obtaining further knowhow from Arnold Bito and Klaus Richter at the Academy of Sciences in Salzburg on working with phage λgt11. The expression library was going to be screened, in a manner identical to immunoblots—with sera from birch pollen allergic patients—a combination of techniques that had not been previously used.

Phage λgt11 clones containing Bet v 1-encoding cDNAs, and thus producing recombinant Bet v 1 in infected *E. coli* cells, were first identified on 3 July 1988. The full cDNA sequence of Bet v 1 was published in the EMBO Journal in 1989 [6], representing the most abundant isoform in birch pollen, called Bet v 1.0101. Thus, Bet v 1 became the first cloned plant allergen and also the first allergenic PR-10-like protein that was published worldwide. Its sequence was similar to the N-terminal peptide sequences of the pollen allergens of hazel, alder and hornbeam, trees belonging to the order of Fagales and, therefore, closely related to birch [7]. What came as a surprise was that Bet v 1 also displayed a 55% sequence identity with a pea disease resistance response gene. Pea and birch being taxonomically quite distant, this was the first indication of a gene family whose founding member must have existed a long time ago. In 1991, the cDNA sequence and recombinant protein of the alder pollen allergen Aln g 1 was published [8], followed in 1993 by the major allergen of hazel pollen, Cor a 1 [9]. This publication describes the identification of four cDNA clones whose open reading frames coded for different isoforms of the major hazel pollen allergen. Interestingly, recombinant proteins of these four isoforms displayed differing IgE binding capacities. In 1995, the cDNAs and recombinant proteins of the major allergens of apple, Mal d 1 [10], and of celery, Api g 1 [11], joined the ranks of allergenic PR-10-like proteins, thus firmly establishing the presence of members of this gene family in the plant kingdom.

## 2. The PR-10-like Family of Allergenic Proteins

In 1980, van Lonn and colleagues defined pathogenesis-related proteins (PR proteins) as “proteins encoded by the host plant but induced only in pathological or related situations” and suggested the first groupings of such PR proteins into families [12]. Currently, 17 such families are described whose members are key components of the plant innate immune system [13]. Plants use PR proteins in inducible defense responses to combat various biotic and abiotic stresses. In contrast, Bet v 1, which is related by sequence to the PR-10 family of proteins, is constitutively expressed in pollen at rather high concentrations. Hence, the correct term for Bet v 1 and its homologous proteins is PR-10-like proteins. The Bet v 1 homologs are slightly acidic small (154–163 amino acid residues) and predominantly cytoplasmic proteins with molecular masses of around 17 kDa. In general, these proteins are susceptible to gastric digestion [14] and heat processing results in a loss of the native protein fold via denaturation, oligomerization and precipitation along with a subsequent reduction in IgE recognition [15]. Some exceptions exist; for example, the allergenicity of the Bet v 1 homolog from carrot, Dau c 1, is not destroyed by cooking [16].

Birch flowers in spring, and its pollen is one of the most common causes of IgE-mediated allergies in Northern and Central Europe as well as in North America [17]. The major sensitizing allergen present in birch pollen is Bet v 1 which is regarded as a marker allergen for a primary sensitization to pollen of birch and other Fagales trees (e.g., alder, hazel, hornbeam, beech, oak). However, allergic reactions to Fagales pollen can be initiated independently by PR-10-like proteins from pollen of all members of the Betulaceae and Fagaceae families (Table 1). However, 25% of the IgE-binding epitopes of PR-10-like allergens from the pollen of trees of the Betuloideae and Coryloideae families are unique to these subfamilies, while pollen allergens from the Fagaceae are generally cross-reactive [18].

Bet v 1 and its homologs in pollen are considered important inducers of birch pollen-associated plant food allergies [17]. The most frequently observed clinical entity, the oral allergy syndrome (OAS) [24], is caused by IgE antibodies that cross-react between Bet v 1 and its homologs in fruits, nuts, seeds and vegetables. Homologs of Bet v 1 have been identified in a wide range of plant foods (Table 2). Hence, Bet v 1-allergic patients are at risk to even react to novel foods without prior exposure.

In contrast to Bet v 1, Bet v 1-related food allergens are in general unable to act as primary sensitizers of predisposed individuals. The major reason for the very low ability of most plant food Bet v 1 homologs to induce sensitization is their high susceptibility to gastric digestion [14]. In addition to the not life-threatening symptoms of the OAS (e.g., itching, redness and tearing of the eyes, itch in the nose and oropharynx, sneezing, runny or stuffy nose, wheezing), severe reactions to Gly m 4, the Bet v 1 homolog from soybean, have been observed in a subpopulation of Bet v 1-allergic individuals [24,43].

## 3. Natural Ligands of PR-10-like Allergenic Proteins and Their Biological Functions

**Figure 1:**
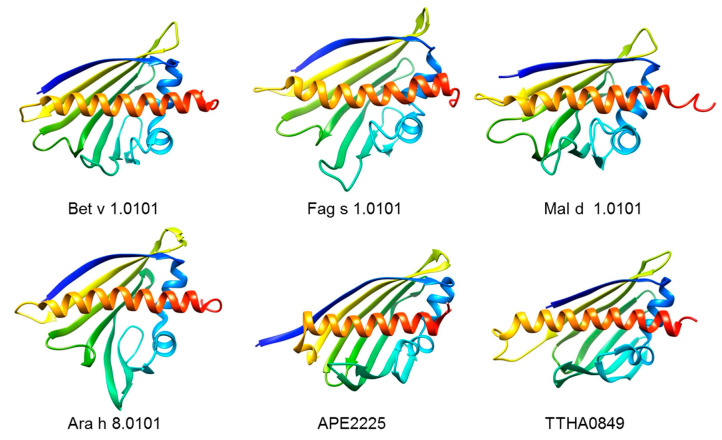
Ribbon representations of birch pollen Bet v 1 (PDB 4A88) and homologs from beech pollen (Fag s 1; PDB 6ALK), apple (Mal d 1; PDB 5MMU), peanut (Ara h 8; PDB 4M9B), the hyperthermophilic archaeon *Aeropyrum pernix* (APE2225; 2NS9) and the extremely thermophilic bacterium *Thermus thermophilus* (TTHA0849; PDB 2D4R) rainbow-colored from blue at the N-terminus to red at the C-terminus. The 3D images were created with the molecular modeling system UCSF ChimeraX (https://www.rbvi.ucsf.edu/chimerax/, accessed on 9 June 2023).

The three-dimensional structure of the major birch pollen allergen Bet v 1 was the first experimentally determined structure of a clinically important major inhalant allergen [44]. The so-called Bet v 1 fold (Figure 1) consists of a seven-stranded anti-parallel beta-sheet that wraps around a 25 residue-long C-terminal alpha-helix. The beta-sheet and the C-terminal part of the long a-helix are separated be two consecutive alpha-helices. The main structural feature of the Bet v 1 fold is a long forked cavity that penetrates the whole protein and that is solvent accessible via three openings to the protein’s surface. The volume of the cavity is about 1500 Å^3^ and its surface is predominantly hydrophobic. This hydrophobic cavity has the ability to bind a variety of experimental and physiologic ligands [45,46]. A detailed description of ligand classes interacting with PR-10 allergens is provided in the 2020 review by Aglas and colleagues [47]. McBride and colleagues chose various polyphenols, fatty acids, phenols, plant hormones, and one plant and one animal sterol to routinely screen for ligand binding characteristics of PR-10-like allergens [48]. However, studies on the physiologic natural ligands of PR-10-like allergens are still limited.

The first physiologic ligand of a PR-10-like protein was determined for Bet v 1. Quercetin-3-*O*-sophoroside (Q3OS), a glycosylated flavonol, was found as a natural ligand bound to Bet v 1 isolated from mature birch pollen [49]. Flavonoids contribute to pigment formation in flowering plants, are involved in plant hormone signaling, facilitate pollen tube formation and protect pollen DNA from UV radiation [50,51]. Flavonoids are stored in pollen as glycosylated precursors and, during the rehydration of the pollen grain, are processed into their active form by pollen glycosyltransferases. At this time point, Q3OS would need to be displaced from the Bet v 1-Q3OS complex to become accessible for deglycosylation. In solution, Bet v 1.0101 is conformationally heterogeneous and cannot be represented by a single structure [52]. NMR relaxation data suggest that structural dynamics are fundamental for ligand access to the protein interior. Complex formation then leads to a significant rigidification of the protein, along with a compaction of its three-dimensional structure, which is observed in the crystalized protein. Ligand binding stabilizes the conformation of Bet v 1, resulting in an increased melting point as well as drastically increased resistance towards endo-/lysosomal proteolysis [53]. The increased stability could hinder optimal proteolytic processing of the allergen, which would favor the development of a Th2 immune response. Analyses of human IgE binding on Bet v 1 in mediator release assays revealed that ligand-bound allergen-induced degranulation at lower concentrations. However, in basophil activation tests with human basophils, ligand-binding did not show this effect [53]. Phytoprostane E1 (PPE1) was identified as another physiologic ligand of Bet v 1 [54]. PPE1 interacts with Bet v 1, increasing its stability to proteolytic degradation. Pollen-derived PPE1 interacts with Bet v 1 with high affinity, increasing its stability and attenuating its degradation by processing by lysosomal cathepsin S. PPE1 also inhibited lysosomal cathepsins by blocking their catalytic cysteines. In addition, processing of Bet v 1.0101 and a hypoallergenic isoform differed distinctly, resulting in low- or high-density class II MHC loading and subsequently in Th2 and Th1 polarization, respectively [55]. Binding of glycosylated flavonoids to Bet v 1 isoforms is governed by the sugar moiety, and various isoforms show individual and highly specific binding behaviors for the different ligands [56]. It is tempting to speculate that the binding of phytoprostanes is also isoform-specific, resulting in the observed differences in Bet v 1 isoform allergenicity.

Natural Cor a 1 was extracted from mature hazel pollen, and its ligand was identified as quercetin-3-O-(2-O-β-D-glucopyranosyl)-β-D-galactopyranoside (Q3O-(Glc)-Gal) [57]. Similarly to nBet v 1, nCor a 1 is composed of different isoallergens and variants. The authors of this study were able to confirm the presence of the variants Cor a 1.0103 and Cor a 1.0104 on the basis of variant-specific tryptic peptides. Furthermore, four known Cor a 1 variants previously detected in hazel pollen and one isoform, Cor a 1.0401, detected only in hazel nuts [30] were analyzed as recombinant proteins for binding the ligand Q3O-(Glc)-Gal. Interestingly, Q3O-(Glc)-Gal was identified as a natural ligand of Cor a 1.0401 [57]. It was further demonstrated that Cor a 1.0401 and Bet v 1.0101 exhibited highly selective binding for their specific ligand but not for the respective ligand of the other allergen. A hypothesis of the role of Q3O-(Glc)-Gal in the unusual reproductive biology of hazel is presented by the authors of the study. The ligand is released from pollen, bound by Cor a 1.0401 present in the stigma, and hence in the nut, to prevent premature deglycosylation, and then only released after several months to be converted into quercetin to assist the formation of the secondary pollen tube.

Emanuelsson and co-workers reported that fruits of colorless white strawberry cultivars showed very low levels of Fra a 1 expression in contrast to red-colored fruits, as well as a downregulation of several enzymes in the pathway for the biosynthesis of flavonoids to which the red color pelargonidin belongs [58]. Casañal and coworkers presented crystallographic structures of Fra a 1 isoforms in complex with the naturally occurring flavonoid catechin, providing for the first time, a molecular basis for the function of these proteins in flavonoid biosynthesis [59]. The authors conclude that Fra a 1 proteins could act as transporters or “chemical chaperones” binding to flavonoid intermediates and making them available to processing enzymes, or they function as cytosolic transporters of flavonoids from the endoplasmic reticulum to other cellular membranes.

## 4. The Bet v 1-like Superfamily of Proteins

The determination of the Bet v 1 structure [44] paved the way for the search and definition of the Bet v 1-like superfamily. Radauer and colleagues used psc++, an improved version of the ProSup structural alignment program [60] to search the Protein Data Bank for structural homologs of Bet v 1 [61]. The resulting structures were then classified into eleven families, of which the Bet v 1 family was one. Today the so called Bet_v_1_like set, which corresponds to the original Pfam Clan CL0209, encompasses 25 families (https://www.ebi.ac.uk/interpro/set/pfam/CL0209/; accessed on 19 June 2023). The underscores in the allergen designation were added by the InterPro database. This designation is not in accordance with the official designation that was assigned by the WHO/IUIS Allergen Nomenclature Sub-Committee (http://allergen.org/viewallergen.php?aid=129, accessed on 19 June 2023). The most widely distributed families of the Bet v 1 superfamily were the polyketide cyclase family and the AHA1 (activator of Hsp90 ATPase homolog 1) family. Members of both families are found in bacteria, archaea and eukaryotes. The ring hydroxylases and the CoxG (named after the *cox g* gene encoding a subunit of the carbon monoxide dehydrogenase) families are widely distributed in bacteria and archaea and the StART (steroidogenic acute regulatory protein-related lipid transfer) family in bacteria and eukaryotes. The phosphatidylinositol transfer proteins are found only in eukaryotes, and members of the Bet v 1 family are exclusively present in plants.

The Bet v 1 family was classified into 11 subfamilies, including 9 from plants and 2 from bacteria [61]. The largest subfamily is the dicot PR-10 subfamily, which contains genuine PR proteins whose expression is upregulated upon pathogen infection, wounding or by abiotic stress, and PR-10-like proteins whose expression is developmentally regulated. The vast majority of allergens are found in the PR-10 subfamily. Allergenic members of other subfamilies were only rarely described and include the mung bean allergen Vig r 6 [34], a member of the subfamily of the cytokinin-specific binding proteins, and the kiwi allergen Act d 11 [26], a member of the major latex protein/ripening-related protein subfamily. So far, this limits the allergens to 3 of 11 subfamilies and to 1 of 25 families of the Bet v 1-like superfamily. The reason that allergenic members of the Bet v 1-like superfamily were found almost exclusively in the PR-10 subfamily is probably linked to the specific ligands they harbor (see Section 3 above), to other biologically active matrix components of the specific plant tissues and the ease or absence of exposure.

A truly ancient protein possessing the Bet v 1 fold must have existed as the last universal common ancestor of this superfamily, giving rise to proteins with diverse functions by insertion of additional structural elements or by fusion to other domains. During evolution, sequence similarities decreased to very low values, making sequence-based searches for Bet v 1 homologs unfeasible. The APE2225 (PDB 2NS9), a CoxG family member from the archaeon *Aeropyrum pernix* K1, has a fold identical to Bet v 1 (Figure 1) but only 14% sequence identity. *A. pernix* K1 is an aerobic hyperthermophilic archaeon isolated from a coastal hydrothermal vent at Kodakara-Jima Island, Japan [62]. This archaeon grows optimally at 90 to 95 °C, pH 7.0, and a salinity of 3.5%, which indicates a high stability of its Bet v 1 fold, a feature that was lost once the Bet v 1 predecessor gene moved into land plants. Recently, a bacterial Bet v 1-related protein, possibly a member of the polyketide cyclase family, TTHA0849 (Figure 1) [63] from *Thermus thermophilus* served as a non-allergenic scaffold to create chimeric proteins by grafting individual epitope-sized, contiguous surface patches of the allergen Bet v 1 onto its surface [64]. These chimeras were then used to determine patient-specific patterns of epitope recognition by IgE antibodies. The norcoclaurine synthases (NCS) form another subfamily of the Bet v 1 family [61]. The NCS from the plant meadow rue (*Thalictrum flavum*), a structural homolog of Bet v 1, does not bind Bet v 1-reactive IgE and was therefore used as another scaffold for grafting of a Bet v 1-specific IgE epitope [65].

## 5. The Yeast Connection

Yeast represents a highly useful genetic model system due to its easily manipulated genome and easily manageable methods for the functional analysis of gene products. Thus, yeast is easily suited to identify biological functions of new and unknown eukaryotic protein-coding genes. We have therefore searched for Bet v 1 homologs in the yeast genome and found a family of sterol transfer proteins recently described in the literature.

Sterols play a key role in regulating the fluidity and barrier function of plasma membranes in eukaryotic cells [66]. Sterol transport proteins distribute sterols from their point of synthesis in the endoplasmic reticulum (ER) to their exact subcellular localization. The ability to solubilize lipids into aqueous solutions is a general property of steroidogenic acute regulatory protein-related lipid transfer (StART) -like domains. Gatta and colleagues have identified a family of StART domain containing proteins that are anchored at membrane contact sites and that transport sterols from the ER to the plasma membrane [67]. The yeast *Saccharomyces cerevisiae* possesses six such proteins with StARkin domains [67,68,69] of which four (Lam1–Lam4) are anchored to the ER membrane. The StARkin domain (the name is derived from kin of steroidogenic acute regulatory protein) is an α/β helix-grip-fold structure with a deep hydrophobic pocket [70]. The major birch pollen allergen Bet v 1 represents the first described StARkin domain [44]. StARkin domains were used to define a large superfamily of proteins, which is also referred to as the Bet v 1-like superfamily [61] or START domain superfamily [71]. The name StARkin was proposed as a more inclusive name for this superfamily [72].

The name Lam is derived from “lipid transport proteins anchored at membrane contact sites”. They are integral membrane proteins anchored into the ER membrane by a C-terminal transmembrane helix [72]. The yeast proteins Lam1 and Lam3 have a single StARkin domain, whereas Lam2 and Lam4 each have two StARkin domains. Jentsch and colleagues carried out a structure-function analysis of the second StARkin domain of Lam4 (Lam4 SD2), showing that Lam4 SD2 undergoes conformational changes upon binding sterol and that it catalyzes sterol transport between vesicles in vitro [73]. Crystal structures of Lam4 SD2 with and without bound 25-hydroxycholesterol were also obtained. Tong and colleagues obtained crystal structures of both StARkin domains of Lam2 and Lam4 in the apo form and of Lam2 SD2 in complex with ergosterol [74].

**Figure 2:**
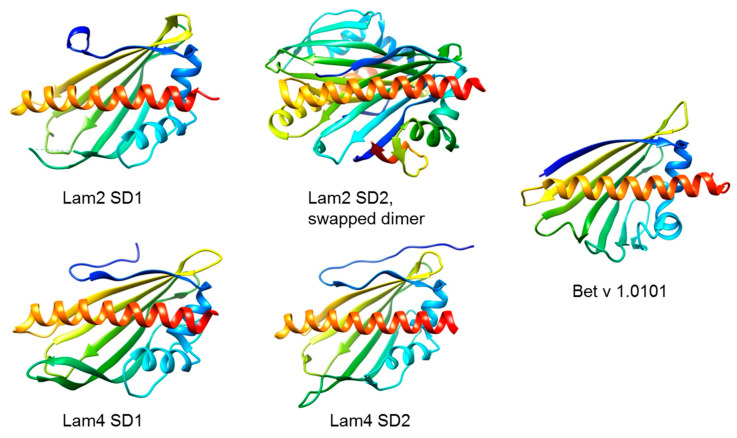
Ribbon representations of Bet v 1 (PDB 4A88) homologous structures from the yeast *Saccharomyces cerevisiae*. The StARkin domains of Lam2 (StARkin domain 1, PDB 5YQI; StARkin domain 2 as a swapped dimer, PDB 5YQQ) and of Lam4 (StARkin domain 1, PDB 5YQJ; StARkin domain 2, PDB 5YQP) are shown. The structures are rainbow-colored from blue at the N-terminus to red at the C-terminus. The 3D images were created with the molecular modeling system UCSF ChimeraX (https://www.rbvi.ucsf.edu/chimerax/, accessed on 19 June 2023).

**Figure 3:**
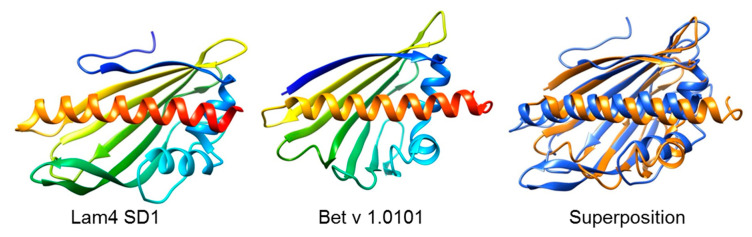
Superposition of the ribbon structure of Bet v 1.0101 (PDB 4A88, aa2-160) in orange and the structure of the StARkin domain 1 of the yeast Lam4 protein (PDB 5YQJ, aa749-929). The 3D images were created with the molecular modeling system UCSF ChimeraX (https://www.rbvi.ucsf.edu/chimerax/, accessed on 19 June 2023).

To identify proteins with similar structures to Bet v 1.0101, we used the online available Vector Alignment Search Tool+ (https://structure.ncbi.nlm.nih.gov/Structure/VAST/vast.shtml, accessed on 13 July 2023) [75,76]. As a bait, NMR data (PDB: 6R3C) of the allergen were chosen [57]. Figure 2 shows ribbon representations of Bet v 1.0101 and the StARkin domains of Lam2 and Lam4, whereby Lam2 SD2 is only available as a swapped dimer. Figure 3 shows an overlay of Bet v 1.0101 and the first StARkin domain of Lam4 (PDB 5YQJ) [74], one of the results of the VAST+ analysis.

## 6. Conclusions

The research on Bet v 1 has spanned five decades. It started in the 1980s with an allergy-driven focus and has expanded into numerous and highly diversified studies of the functions of the members of the Bet v 1-like/StARkin superfamily of proteins. The first described StARkin domain was Bet v 1. Identification of ligands of Bet v 1 and its homologs offer the first insights into the varied functions of these proteins. However, information on ligands and functions of the various isoforms of Bet v 1 in birch pollen is still missing, as it is unclear why there are so many isoforms present. The Bet v 1 scaffold is a very old and versatile one and has been used for a wide variety of functions. We do not believe that the Bet v 1 homologs present in birch or in other organisms are the result of convergent evolution. Rather, it is a very useful scaffold that was conserved and used in many different ways. The Bet v 1-like/StARkin superfamily keeps growing, and new members are still being discovered.

## Figures and Tables

**Table 1:** Bet v 1-homologous pollen allergens of trees of the order Fagales.

Plant Family	Subfamily	Allergen Source	Allergen	References	UniProt/PDB
Betulaceae	Betuloideae	Birch (*Betula pendula*)	Bet v 1.0101	[6]	P15494/4A88
		Alder (*Alnus glutinosa*)	Aln g 1.0101	[8]	P38948
	Coryloideae	Hazel (*Corylus avellana*)	Cor a 1.0101	[9]	Q09407
		Hornbeam (*Carpinus betulus*)	Car b 1.0101	[19]	P38949
		Hop-hornbeam (*Ostrya carpinifolia*)	Ost c 1.0101	[18]	E2GL17
Fagaceae	Fagoideae	Beech (*Fagus silvatica*)	Fag s 1.0101	[18]	B7TWE6/6ALK
	Quercoideae	Oak (*Quercus alba*)	Que a 1.0201	[20]	B6RQS1
		Sawtooth oak *(Quercus acutissima*)	Que ac 1.0101	[21]	GenBank QOL10866.1
		Holly oak *(Quercus ilex)*	Que i 1.0101	[22]	A0A7D0TA82
		Mongolian oak (*Quercus mongolica)*	Que m 1.0101	[23]	GenBank AUH28179
	Castanoideae	Chestnut (*Castanea sativa*)	Cas s 1.0101	[18]	B7TWE3

**Table 2:** Plant food allergenic PR-10-like proteins.

Plant Family	Allergen Source	Allergen	References	UniProt/PDB
Actinidiaceae	Golden kiwifruit (*Actinidia chinensis*)	Act c 8.0101	[25]	D1YSM4
	Green kiwifruit (*Actinidia deliciosa*)	Act d 8.0101 Act d 11.0101	[25] [26]	D1YSM5 P85524/4IGV
Anacardiaceae	Mango (*Mangifera indica*)	Man i 2.0101	[27]	GenBank UYO79702.1
Apiaceae	Celery (*Apium graveolens*)	Api g 1.0101	[11]	P49372/2BK0
	Carrot (*Daucus carota*)	Dau c 1.0103	[28]	O04298/2WQL
Cannabaceae	Indian hemp (*Cannabis sativa*)	Can s 5.0101	[29]	I6XT51
Corylaceae	Hazelnut (*Corylus avellana*)	Cor a 1.0401	[30]	Q9SWR4/6GQ9, 6Y3H
Fabaceae	Peanut (*Arachis hypogaea*)	Ara h 8.0101	[31]	Q6VT83/4M9B
	Soybean (*Glycine max*)	Gly m 4.0101	[32]	P26987/2K7H
	Mung bean (*Vigna radiata*)	Vig r 1.0101 Vig r 6.0101	[33] [34]	Q2VU97 A0A1S3THR8/2FLH, 3C0V
Juglandaceae	English walnut (*Juglans regia*)	Jug r 5.0101	[35]	GenBank Acc. No. KX034087.1
Rosaceae	Strawberry (*Fragaria x ananassa*)	Fra a 1.0101	[36]	Q5ULZ4/6ST8
	Apple (*Malus domestica*)	Mal d 1.0101	[10]	P43211/5MMU
	Apricot (*Prunus armeniaca*)	Pru ar 1.0101	unpublished	O50001
	Cherry (*Prunus avium*)	Pru av 1.0101	[37]	O24248/1E09, 1H20
	Almond (*Prunus dulcis*)	Pru du 1.0101	[38]	B6CQS9
	Peach (*Prunus persica*)	Pru p 1.0101	[39]	Q2I6V8/6Z98
	Pear (*Pyrus communis*)	Pyr c 1.0101	[40]	O65200
	Raspberry (*Rubus idaeus*)	Rub 1 1.0101	[41]	Q0Z8U9
Solanaceae	Tomato (*Solanum lycopersicum*)	Sola l 4.0101	[42]	K4CWC5

## References

[1] Breiteneder H., Hendler P.N., Kraft D. (2020). Legends of allergy and immunology: Clemens von Pirquet. Allergy.

[2] Breiteneder H. (2019). Legends of allergy/immunology: Dietrich Kraft. Allergy.

[3] King T.P., Hoffman D., Lowenstein H., Marsh D.G., Platts-Mills T.A., Thomas W. (1995). Allergen nomenclature. Allergy.

[4] Breiteneder H., Seiser C., Loffelhardt W., Michalowski C., Bohnert H.J. (1988). Physical map and protein gene map of cyanelle DNA from the second known isolate of *Cyanophora paradoxa* (Kies-strain). Curr. Genet..

[5] Breiteneder H., Hassfeld W., Pettenburger K., Jarolim E., Breitenbach M., Rumpold H., Kraft D., Scheiner O. (1988). Isolation and characterization of messenger RNA from male inflorescences and pollen of the white birch (*Betula verrucosa*). Int. Arch. Allergy Appl. Immunol..

[6] Breiteneder H., Pettenburger K., Bito A., Valenta R., Kraft D., Rumpold H., Scheiner O., Breitenbach M. (1989). The gene coding for the major birch pollen allergen Betv1, is highly homologous to a pea disease resistance response gene. EMBO J..

[7] Valenta R., Breiteneder H., Petternburger K., Breitenbach M., Rumpold H., Kraft D., Scheiner O. (1991). Homology of the major birch-pollen allergen, *Bet v* I, with the major pollen allergens of alder, hazel, and hornbeam at the nucleic acid level as determined by cross-hybridization. J. Allergy Clin. Immunol..

[8] Breiteneder H., Ferreira F., Reikerstorfer A., Duchene M., Valenta R., Hoffmann-Sommergruber K., Ebner C., Breitenbach M., Kraft D., Scheiner O. (1992). Complementary DNA cloning and expression in *Escherichia coli* of Aln g I, the major allergen in pollen of alder (*Alnus glutinosa*). J. Allergy Clin. Immunol..

[9] Breiteneder H., Ferreira F., Hoffmann-Sommergruber K., Ebner C., Breitenbach M., Rumpold H., Kraft D., Scheiner O. (1993). Four recombinant isoforms of *Cor a* I, the major allergen of hazel pollen, show different IgE-binding properties. Eur. J. Biochem..

[10] Vanek-Krebitz M., Hoffmann-Sommergruber K., Laimer da Camara Machado M., Susani M., Ebner C., Kraft D., Scheiner O., Breiteneder H. (1995). Cloning and sequencing of Mal d 1, the major allergen from apple (*Malus domestica*), and its immunological relationship to Bet v 1, the major birch pollen allergen. Biochem. Biophys. Res. Commun..

[11] Breiteneder H., Hoffmann-Sommergruber K., O’Riordain G., Susani M., Ahorn H., Ebner C., Kraft D., Scheiner O. (1995). Molecular characterization of Api g 1, the major allergen of celery (*Apium graveolens*), and its immunological and structural relationships to a group of 17-kDa tree pollen allergens. Eur. J. Biochem..

[12] Antoniw J.F., Ritter C.E., Pierpoint W.S., Vanloon L.C. (1980). Comparison of 3 pathogenesis-related proteins from plants of 2 cultivars of tobacco infected with TMV. J. Gen. Virol..

[13] Ali S., Ganai B.A., Kamili A.N., Bhat A.A., Mir Z.A., Bhat J.A., Tyagi A., Islam S.T., Mushtaq M., Yadav P. (2018). Pathogenesis-related proteins and peptides as promising tools for engineering plants with multiple stress tolerance. Microbiol. Res..

[14] Sancho A.I., Wangorsch A., Jensen B.M., Watson A., Alexeev Y., Johnson P.E., Mackie A.R., Neubauer A., Reese G., Ballmer-Weber B. (2011). Responsiveness of the major birch allergen Bet v 1 scaffold to the gastric environment: Impact on structure and allergenic activity. Mol. Nutr. Food Res..

[15] Rib-Schmidt C., Riedl P., Meisinger V., Schwaben L., Schulenborg T., Reuter A., Schiller D., Seutter von Loetzen C., Rosch P. (2018). pH and heat resistance of the major celery allergen Api g 1. Mol. Nutr. Food Res..

[16] Jacob T., Vogel L., Reuter A., Wangorsch A., Kring C., Mahler V., Wohrl B.M. (2020). Food processing does not abolish the allergenicity of the carrot allergen Dau c 1: Influence of pH, temperature, and the food matrix. Mol. Nutr. Food Res..

[17] Dramburg S., Hilger C., Santos A.F., de Las Vecillas L., Aalberse R.C., Acevedo N., Aglas L., Altmann F., Arruda K.L., Asero R. (2023). EAACI Molecular Allergology User’s Guide 2.0. Pediatr. Allergy Immunol..

[18] Hauser M., Asam C., Himly M., Palazzo P., Voltolini S., Montanari C., Briza P., Bernardi M.L., Mari A., Ferreira F. (2011). Bet v 1-like pollen allergens of multiple Fagales species can sensitize atopic individuals. Clin. Exp. Allergy.

[19] Larsen J.N., Stroman P., Ipsen H. (1992). PCR based cloning and sequencing of isogenes encoding the tree pollen major allergen Car b I from *Carpinus betulus*, hornbeam. Mol. Immunol..

[20] Wallner M., Erler A., Hauser M., Klinglmayr E., Gadermaier G., Vogel L., Mari A., Bohle B., Briza P., Ferreira F. (2009). Immunologic characterization of isoforms of Car b 1 and Que a 1, the major hornbeam and oak pollen allergens. Allergy.

[21] Jeong K.Y., Lee J., Sang M.K., Lee Y.S., Park K.H., Lee J.H., Park J.W. (2021). Sensitization profile to sawtooth oak component allergens and their clinical implications. J. Clin. Lab. Anal..

[22] Pedrosa M., Guerrero-Sanchez V.M., Canales-Bueno N., Loli-Ausejo D., Castillejo M.A., Quirce S., Jorrin-Novo J.V., Rodriguez-Perez R. (2020). Quercus ilex pollen allergen, Que i 1, responsible for pollen food allergy syndrome caused by fruits in Spanish allergic patients. Clin. Exp. Allergy.

[23] Lee J.Y., Yang M., Jeong K.Y., Sim D.W., Park J.H., Park K.H., Lee J.H., Park J.W. (2017). Characterization of a major allergen from Mongolian oak, *Quercus mongolica*, a dominant species of oak in Korea. Int. Arch. Allergy Immunol..

[24] Vieths S., Scheurer S., Ballmer-Weber B. (2002). Current understanding of cross-reactivity of food allergens and pollen. Ann. N. Y. Acad. Sci..

[25] Oberhuber C., Bulley S.M., Ballmer-Weber B.K., Bublin M., Gaier S., DeWitt A.M., Briza P., Hofstetter G., Lidholm J., Vieths S. (2008). Characterization of Bet v 1-related allergens from kiwifruit relevant for patients with combined kiwifruit and birch pollen allergy. Mol. Nutr. Food Res..

[26] D’Avino R., Bernardi M.L., Wallner M., Palazzo P., Camardella L., Tuppo L., Alessandri C., Breiteneder H., Ferreira F., Ciardiello M.A. (2011). Kiwifruit Act d 11 is the first member of the ripening-related protein family identified as an allergen. Allergy.

[27] Zhao L., Xie H., Wang X., Liu M., Ma T., Fu W., Wang Y., Wu D., Feng Y., Liu Y. (2023). Molecular characterization of allergens and component-resolved diagnosis of IgE-mediated mango fruit allergy. Allergy.

[28] Hoffmann-Sommergruber K., O’Riordain G., Ahorn H., Ebner C., Laimer Da Camara Machado M., Puhringer H., Scheiner O., Breiteneder H. (1999). Molecular characterization of Dau c 1, the Bet v 1 homologous protein from carrot and its cross-reactivity with Bet v 1 and Api g 1. Clin. Exp. Allergy.

[29] Ebo D.G., Decuyper I.I., Rihs H.P., Mertens C., Van Gasse A.L., van der Poorten M.M., De Puysseleyr L., Faber M.A., Hagendorens M.M., Bridts C.H. (2021). IgE-binding and mast cell-activating capacity of the homologue of the major birch pollen allergen and profilin from Cannabis sativa. J. Allergy Clin. Immunol. Pract..

[30] Luttkopf D., Muller U., Skov P.S., Ballmer-Weber B.K., Wuthrich B., Skamstrup Hansen K., Poulsen L.K., Kastner M., Haustein D., Vieths S. (2002). Comparison of four variants of a major allergen in hazelnut (*Corylus avellana*) Cor a 1.04 with the major hazel pollen allergen Cor a 1.01. Mol. Immunol..

[31] Mittag D., Akkerdaas J., Ballmer-Weber B.K., Vogel L., Wensing M., Becker W.M., Koppelman S.J., Knulst A.C., Helbling A., Hefle S.L. (2004). Ara h 8, a Bet v 1-homologous allergen from peanut, is a major allergen in patients with combined birch pollen and peanut allergy. J. Allergy Clin. Immunol..

[32] Crowell D.N., John M.E., Russell D., Amasino R.M. (1992). Characterization of a stress-induced, developmentally regulated gene family from soybean. Plant Mol. Biol..

[33] Mittag D., Vieths S., Vogel L., Wagner-Loew D., Starke A., Hunziker P., Becker W.M., Ballmer-Weber B.K. (2005). Birch pollen-related food allergy to legumes: Identification and characterization of the Bet v 1 homologue in mungbean (*Vigna radiata*), Vig r 1. Clin. Exp. Allergy.

[34] Guhsl E.E., Hofstetter G., Hemmer W., Ebner C., Vieths S., Vogel L., Breiteneder H., Radauer C. (2014). Vig r 6, the cytokinin-specific binding protein from mung bean (Vigna radiata) sprouts, cross-reacts with Bet v 1-related allergens and binds IgE from birch pollen allergic patients’ sera. Mol. Nutr. Food Res..

[35] Wangorsch A., Jamin A., Lidholm J., Grani N., Lang C., Ballmer-Weber B., Vieths S., Scheurer S. (2017). Identification and implication of an allergenic PR-10 protein from walnut in birch pollen associated walnut allergy. Mol. Nutr. Food Res..

[36] Musidlowska-Persson A., Alm R., Emanuelsson C. (2007). Cloning and sequencing of the Bet v 1-homologous allergen Fra a 1 in strawberry *(Fragaria ananassa*) shows the presence of an intron and little variability in amino acid sequence. Mol. Immunol..

[37] Scheurer S., Pastorello E.A., Wangorsch A., Kastner M., Haustein D., Vieths S. (2001). Recombinant allergens Pru av 1 and Pru av 4 and a newly identified lipid transfer protein in the in vitro diagnosis of cherry allergy. J. Allergy Clin. Immunol..

[38] Kabasser S., Crvenjak N., Schmalz S., Kalic T., Hafner C., Dubiela P., Kucharczyk A., Bazan-Socha S., Lukaszyk M., Breiteneder H. (2022). Pru du 1, the Bet v 1-homologue from almond, is a major allergen in patients with birch pollen associated almond allergy. Clin. Transl. Allergy.

[39] Wisniewski M., Bassett C., Arora R. (2004). Distribution and partial characterization of seasonally expressed proteins in different aged shoots and roots of ‘Loring’ peach (*Prunus persica*). Tree Physiol..

[40] Karamloo F., Scheurer S., Wangorsch A., May S., Haustein D., Vieths S. (2001). Pyr c 1, the major allergen from pear (*Pyrus communis*), is a new member of the Bet v 1 allergen family. J. Chromatogr. B Biomed. Sci. Appl..

[41] Marzban G., Herndl A., Kolarich D., Maghuly F., Mansfeld A., Hemmer W., Katinger H., Laimer M. (2008). Identification of four IgE-reactive proteins in raspberry *(Rubus ideaeus* L.). Mol. Nutr. Food Res..

[42] Wangorsch A., Jamin A., Foetisch K., Malczyk A., Reuter A., Vierecke S., Schulke S., Bartel D., Mahler V., Lidholm J. (2015). Identification of Sola l 4 as Bet v 1 homologous pathogenesis related-10 allergen in tomato fruits. Mol. Nutr. Food Res..

[43] Kleine-Tebbe J., Vogel L., Crowell D.N., Haustein U.F., Vieths S. (2002). Severe oral allergy syndrome and anaphylactic reactions caused by a Bet v 1- related PR-10 protein in soybean, SAM22. J. Allergy Clin. Immunol..

[44] Gajhede M., Osmark P., Poulsen F.M., Ipsen H., Larsen J.N., Joost van Neerven R.J., Schou C., Lowenstein H., Spangfort M.D. (1996). X-ray and NMR structure of Bet v 1, the origin of birch pollen allergy. Nat. Struct. Biol..

[45] Mogensen J.E., Wimmer R., Larsen J.N., Spangfort M.D., Otzen D.E. (2002). The major birch allergen, Bet v 1, shows affinity for a broad spectrum of physiological ligands. J. Biol. Chem..

[46] Kofler S., Asam C., Eckhard U., Wallner M., Ferreira F., Brandstetter H. (2012). Crystallographically mapped ligand binding differs in high and low IgE binding isoforms of birch pollen allergen bet v 1. J. Mol. Biol..

[47] Aglas L., Soh W.T., Kraiem A., Wenger M., Brandstetter H., Ferreira F. (2020). Ligand Binding of PR-10 Proteins with a Particular Focus on the Bet v 1 Allergen Family. Curr. Allergy Asthma Rep..

[48] McBride J.K., Cheng H., Maleki S.J., Hurlburt B.K. (2019). Purification and characterization of pathogenesis related class 10 panallergens. Foods.

[49] Seutter von Loetzen C., Hoffmann T., Hartl M.J., Schweimer K., Schwab W., Rosch P., Hartl-Spiegelhauer O. (2014). Secret of the major birch pollen allergen Bet v 1: Identification of the physiological ligand. Biochem. J..

[50] Brunetti C., Fini A., Sebastiani F., Gori A., Tattini M. (2018). Modulation of Phytohormone Signaling: A Primary Function of Flavonoids in Plant-Environment Interactions. Front. Plant Sci..

[51] Agati G., Brunetti C., Di Ferdinando M., Ferrini F., Pollastri S., Tattini M. (2013). Functional roles of flavonoids in photoprotection: New evidence, lessons from the past. Plant Physiol. Biochem..

[52] Grutsch S., Fuchs J.E., Freier R., Kofler S., Bibi M., Asam C., Wallner M., Ferreira F., Brandstetter H., Liedl K.R. (2014). Ligand binding modulates the structural dynamics and compactness of the major birch pollen allergen. Biophys. J..

[53] Asam C., Batista A.L., Moraes A.H., de Paula V.S., Almeida F.C., Aglas L., Kitzmuller C., Bohle B., Ebner C., Ferreira F. (2014). Bet v 1—A Trojan horse for small ligands boosting allergic sensitization?. Clin. Exp. Allergy.

[54] Soh W.T., Aglas L., Mueller G.A., Gilles S., Weiss R., Scheiblhofer S., Huber S., Scheidt T., Thompson P.M., Briza P. (2019). Multiple roles of Bet v 1 ligands in allergen stabilization and modulation of endosomal protease activity. Allergy.

[55] Freier R., Dall E., Brandstetter H. (2015). Protease recognition sites in Bet v 1a are cryptic, explaining its slow processing relevant to its allergenicity. Sci. Rep..

[56] Seutter von Loetzen C., Jacob T., Hartl-Spiegelhauer O., Vogel L., Schiller D., Sporlein-Guttler C., Schobert R., Vieths S., Hartl M.J., Rosch P. (2015). Ligand recognition of the major birch pollen allergen Bet v 1 is isoform dependent. PLoS ONE.

[57] Jacob T., von Loetzen C.S., Reuter A., Lacher U., Schiller D., Schobert R., Mahler V., Vieths S., Rosch P., Schweimer K. (2019). Identification of a natural ligand of the hazel allergen Cor a 1. Sci. Rep..

[58] Hjerno K., Alm R., Canback B., Matthiesen R., Trajkovski K., Bjork L., Roepstorff P., Emanuelsson C. (2006). Down-regulation of the strawberry Bet v 1-homologous allergen in concert with the flavonoid biosynthesis pathway in colorless strawberry mutant. Proteomics.

[59] Casanal A., Zander U., Munoz C., Dupeux F., Luque I., Botella M.A., Schwab W., Valpuesta V., Marquez J.A. (2013). The strawberry pathogenesis-related 10 (PR-10) Fra a proteins control flavonoid biosynthesis by binding to metabolic intermediates. J. Biol. Chem..

[60] Lackner P., Koppensteiner W.A., Sippl M.J., Domingues F.S. (2000). ProSup: A refined tool for protein structure alignment. Protein Eng..

[61] Radauer C., Lackner P., Breiteneder H. (2008). The Bet v 1 fold: An ancient, versatile scaffold for binding of large, hydrophobic ligands. BMC Evol. Biol..

[62] Sako Y., Nomura N., Uchida A., Ishida Y., Morii H., Koga Y., Hoaki T., Maruyama T. (1996). Aeropyrum pernix gen. nov., sp. nov., a novel aerobic hyperthermophilic archaeon growing at temperatures up to 100 degrees C. Int. J. Syst. Bacteriol..

[63] Nakabayashi M., Shibata N., Komori H., Ueda Y., Iino H., Ebihara A., Kuramitsu S., Higuchi Y. (2005). Structure of a conserved hypothetical protein, TTHA0849 from Thermus thermophilus HB8, at 2.4 A resolution: A putative member of the StAR-related lipid-transfer (START) domain superfamily. Acta Crystallogr. Sect. F Struct. Biol. Cryst. Commun..

[64] Schmalz S., Mayr V., Shosherova A., Gepp B., Ackerbauer D., Sturm G., Bohle B., Breiteneder H., Radauer C. (2022). Isotype-specific binding patterns of serum antibodies to multiple conformational epitopes of Bet v 1. J. Allergy Clin. Immunol..

[65] Berkner H., Seutter von Loetzen C., Hartl M., Randow S., Gubesch M., Vogel L., Husslik F., Reuter A., Lidholm J., Ballmer-Weber B. (2014). Enlarging the toolbox for allergen epitope definition with an allergen-type model protein. PLoS ONE.

[66] Holthuis J.C., Menon A.K. (2014). Lipid landscapes and pipelines in membrane homeostasis. Nature.

[67] Gatta A.T., Wong L.H., Sere Y.Y., Calderon-Norena D.M., Cockcroft S., Menon A.K., Levine T.P. (2015). A new family of StART domain proteins at membrane contact sites has a role in ER-PM sterol transport. eLife.

[68] Elbaz-Alon Y., Eisenberg-Bord M., Shinder V., Stiller S.B., Shimoni E., Wiedemann N., Geiger T., Schuldiner M. (2015). Lam6 regulates the extent of contacts between organelles. Cell Rep..

[69] Murley A., Sarsam R.D., Toulmay A., Yamada J., Prinz W.A., Nunnari J. (2015). Ltc1 is an ER-localized sterol transporter and a component of ER-mitochondria and ER-vacuole contacts. J. Cell Biol..

[70] Dresden C.E., Ashraf Q., Husbands A.Y. (2021). Diverse regulatory mechanisms of StARkin domains in land plants and mammals. Curr. Opin. Plant Biol..

[71] Iyer L.M., Koonin E.V., Aravind L. (2001). Adaptations of the helix-grip fold for ligand binding and catalysis in the START domain superfamily. Proteins.

[72] Wong L.H., Levine T.P. (2016). Lipid transfer proteins do their thing anchored at membrane contact sites… but what is their thing?. Biochem. Soc. Trans..

[73] Jentsch J.A., Kiburu I., Pandey K., Timme M., Ramlall T., Levkau B., Wu J., Eliezer D., Boudker O., Menon A.K. (2018). Structural basis of sterol binding and transport by a yeast StARkin domain. J. Biol. Chem..

[74] Tong J., Manik M.K., Im Y.J. (2018). Structural basis of sterol recognition and nonvesicular transport by lipid transfer proteins anchored at membrane contact sites. Proc. Natl. Acad. Sci. USA.

[75] Gibrat J.F., Madej T., Bryant S.H. (1996). Surprising similarities in structure comparison. Curr. Opin. Struct. Biol..

[76] Madej T., Marchler-Bauer A., Lanczycki C., Zhang D., Bryant S.H. (2020). Biological assembly comparison with VAST. Methods Mol. Biol..

